# Surgical management of symptomatic cavum septum pellucidum cysts: systematic review of the literature

**DOI:** 10.1007/s10143-020-01447-4

**Published:** 2020-12-19

**Authors:** Alexandre Simonin, Christopher R. P. Lind

**Affiliations:** 1 Department of Neurosurgery, Sir Charles Gairdner Hospital (SCGH), Level 1, Nedlands, WA 6009 Australia; 2 Department of Clinical Neurosciences, Service of Neurosurgery, Lausanne University Hospital (CHUV), Lausanne, Switzerland; 3 Medical School, University of Western Australia, Perth, WA Australia

**Keywords:** Cavum septum pellucidum, Cavum vergae, Endoscopic fenestration

## Abstract

Cavum septum pellucidum (CSP) and cavum vergae (CV) cysts are commonly found incidentally. They are usually asymptomatic but may present with symptoms related to obstructive hydrocephalus. There is no consensus about the management of symptomatic CSP and CV cysts. We present, to the best of our knowledge, the first systematic review of the different treatment options for symptomatic CSP and CV cysts. We conducted a literature review using PubMed database, searching for cases of symptomatic CSP and CV cysts managed surgically, and published until April 2019. Preoperative characteristics, surgical procedure, and postoperative outcome were analyzed using SPSS® software (Statistical Package for Social Sciences, IBM®). We found 54 cases of symptomatic CSP and CV cysts managed surgically (34 males, 20 females, 1.7/1 male to female ratio). Mean age was 24.3 ± 20.1 years. The most common presentation was headaches (34 patients, 62%), followed by psychiatric symptoms (27 patients, 49.1%). Preoperative radiological hydrocephalus was present in 30 patients (54.5%). The most common surgical procedure was endoscopic fenestration (39 patients, 70.9%), followed by shunting (10 patients, 18.2%), open surgery (3 patients, 5.5%), and stereotactic fenestration (1 patient, 1.8%). Complete resolution of symptoms was achieved in 36 patients (65.5%) and partial resolution in 7 patients (12.7%), and symptoms were unchanged in 2 patients. The present review suggests that surgical treatment could provide resolution of the symptoms in most of the cases, regardless of the procedure performed. Although mean follow-up was short among the studies, recurrence rate was low.

## Introduction

Cavum septum pellucidum (CSP) is a common incidental finding, defined as a midline cerebrospinal fluid (CSF) space delimited superiorly by the crus of the fornices and inferiorly by the tela choroidea of the third ventricle [1]. It is anatomically distinct from cavum vergae (CV) which is a CSF space extending posteriorly to the columns of the fornix. However, CSP and CV cysts are used interchangeably in the literature and may co-exist in many cases [1–5]. In this manuscript, we will use the terminology cavum septum pellucidum and vergae (CSP and CV) cyst. Although considered as an incidental finding by most neurosurgeons, they may present with symptoms related to hydrocephalus, like headaches, nausea or vomiting, loss of consciousness, or psychiatric disturbances [1–10]. There is no consensus about the management of symptomatic CSP and CV cysts, and various procedures (endoscopic or stereotactic fenestration, shunting, open fenestration, etc.) have been proposed [1, 3–5, 8, 11–13]. Although most of the authors report good results, there is currently no review of the literature concerning the surgical management of this controversial condition. The aim of the present study is to review the preoperative characteristics, surgical procedures, and postoperative outcome of symptomatic CSP and CV cysts treated surgically and reported in the literature.

## Methods

We conducted a literature review using PubMed database, searching for cases of symptomatic CSP and CV cysts managed surgically and published until April 2019. We used the search term “cavum septum pellucidum” to perform the research. Three hundred and forty-five articles were found, and we considered 37 articles eligible for our study [1–35] based on the criteria that the management of a symptomatic cyst was detailed in the article. Twenty-three articles reported cases managed surgically [1–3, 6, 8, 10, 14, 15, 17, 18, 21–27, 32–35], and a total of 54 patients were finally identified. We created a database using Microsoft Excel® and SPSS® softwares (Statistical Package for Social Sciences, IBM®). Preoperative characteristics (Table 1) were summarized with patient gender, age, clinical presentation, and presence of preoperative hydrocephalus. Management (type of surgical procedure), clinical outcome, radiological outcome, complications, follow-up, and recurrences were also recorded (Table 2). We used SPSS® software (Statistical Package for Social Sciences, IBM®) descriptive statistics to analyze age and follow-up means and standard deviation. Frequency statistics were used to analyze gender (male/female), headaches (yes/no), psychiatric symptoms (yes/no), loss of consciousness (yes/no), seizures (yes/no), nausea/vomiting (yes/no), papilledema (yes/no), preoperative hydrocephalus (yes/no), surgical procedure (endoscopic, stereotactic, open surgery, shunting), type of endoscopic procedure (frontal, parietal, transcavum), shunt (definitive, transient, or none), complications (yes/no), clinical outcome (resolution of symptoms/improvement/unchanged/died/unknown), radiological outcome (significant decrease of the cyst/persistent enlargement/unknown), and recurrences (yes/no). We used an unpaired Student’s *t* test to look for significant differences in clinical presentation and outcome between male and female patients. Unpaired Student’s *t* test was used to look for independent variables between males and females and between children and adults and to identify independent predictors of good clinical outcome (resolved of improved symptoms), recurrence, and good radiological outcome (decrease of the cyst size on postoperative imaging).

**Table 1:** Preoperative characteristics, *n* = 39

Patient	Sex	Age (years)	Clinical presentation	Duration of symptoms (months)	Preoperative hydrocephalus	Reference
1	Male	3	Developmental delay, irritability, macrocephaly		Yes	1)
2	Female	13	Psychiatric symptoms (eating disorder, mood disturbances, anxiety)		No	1)
3	Male	42	Postural headaches, disorders of consciousness		48	2)
4	Male	61	Postural headaches, disorders of consciousness, dizziness, ataxia			2)
5	Male	46	Postural headaches, disorders of consciousness			2)
6	Female	60	Postural headaches		No	2)
7	Male	0.5	Developmental delay		Yes	2)
8	Male	12	Headaches, vomiting	24	Yes	3)
9	Female	13	Macrocephaly, irritability	24	No	3)
10	Female	26	Headaches, blurring of vision		Yes	3)
11	Male	4.5	Impaired mental function with vomiting, seizures			20)
12	Female	1.9	Impaired mental function with hydrocephalus, papilledema, paraparesis	12	Yes	22)
13	Female	2.9	Impaired mental state with hydrocephalus and ataxic gait			23)
14	Male	6	Behavioral disturbance with impaired gait			24)
15	Male	1.5	Hydrocephalus and dyspnea with mental changes			25)
16	Male	13	Headaches, dizziness, behavioral disturbance		Yes	26)
17	Female	23	Headaches, behavioral disturbance	12	Yes	26)
18	Female	19	Epilepsy	2	Yes	26)
19	Male	60	Headaches, unstable gait, papilledema	36	Yes	26)
20	Female	32	Headaches, dizziness, papilledema, behavioral disturbance	12	Yes	26)
21	Male	18	Epilepsy, behavioral disturbance	24	Yes	26)
22	Male	34	Headaches, dizziness, epilepsy, behavioral disturbance	30	Yes	26)
23	Male	3	Headaches, vomiting	26	Yes	26)
24	Male	8	Headaches, behavioral disturbance	16	Yes	26)
25	Male	3	Signs of hydrocephalus	12	Yes	26)
26	Male	8	Headaches, behavioral changes, syncopal attacks		Yes	27)
27	Male	0.5	Headaches, syncopal episodes, neuropsychological disturbances		Yes	28)
28	Male	42	headaches, syncopal episodes, impairment of memory			28)
29	Male	17	Uncontrolled seizures, cognitive impairment		Yes	6)
30	Female	24	Headaches, vomiting, mental dulling, drowsiness		Yes	8)
31	Male	44	Postural headaches		Yes	10)
32	Male	14	Headaches, vomiting, syncope, decreased concentration and attention			14)
33	Male	12	Headaches, disorders of consciousness, rigidity		Yes	15)
34	Male	44	Sudden headaches, loss of consciousness		Yes	17)
35	Male	17	Wilson disease, seizures, cognitive impairment		Yes	18)
36	Male	9	Motor and mental retardation, seizures		No	19)
37	Male	31	Intermittent headaches, nausea, vomiting		Yes	Our series
38	Female	24	Postural headaches, nausea		Yes	Our series
39	Female	22	Headaches, dizziness, nausea, vertigo		Yes	Our series
40	Female	22.5	Explosive headache		24	34)
41	Male	46.8	Explosive headache, nausea, vomiting, behavioral disturbance		4	34)
42	Male	31	Explosive headache		9	34)
43	Male	2	Progressive macrocephaly, unclosed anterior fontanelle, delayed psychomotor development		6	34)
44	Male	13.5	Progressive behavioral deterioration, uncontrollable mood swings, declining school performance		23	34)
45	Female	69	Progressive deterioration of gait, quadriparesis, headache		8	34)
46	Female	9	Learning difficulties, unable to concentrate, emotional changes, memory loss, epilepsy, vomiting, loss of consciousness, declining school performance		72	34)
47	Female	22.8	Headache, vertigo, visual disturbance, memory loss		17	34)
48	Male	12.1	Explosive headache accompanying collapse, visual disturbance		6	34)
49	Male	33	Progressive headache		60	34)
50	Female	11	Progressive headache, mental retardation	60	Yes	35)
51	Female	36	Disturbance of eye-movements, diplopia	0.25	Yes	35)
52	Male	63	Diplopia, headaches, confusion	24	Yes	35)
53	Female	82	Short-term memory deficits and gait instability, falls, urinary incontinence		Yes	37)
54	Female	41	Short-term memory deficits and gait instability, falls, urinary incontinence		Yes	37)

**Table 2:** Management and outcome, *n* = 39

Patient	Management	Clinical outcome	Radiological outcome	Complication	Follow-up (years)	Recurrence
1	Endoscopic fenestration (right parietal)	Improvement of development, unchanged macrocephaly	Decrease of the cyst		0.5	No
2	Endoscopic fenestration (right parietal)	Symptoms unchanged	Decrease of the cyst		2	No
3	Open transcortical approach, fenestration				22	Yes (re-operation)
4	Unsuccessful fenestration, ventriculo-cysto-atrial shunt				15	Yes (shunting)
5						No
6	Stereotactic fenestration				3	No
7	Failed ventriculo-peritoneal shunt	Died	Persistent enlargement	Died	3	No
8	Endoscopic fenestration with navigation	Improved	Decrease of the cyst		4	No
9	Endoscopic fenestration with navigation	Head size stabilized	Decrease of the cyst		3	No
10	Endoscopic fenestration with navigation	Improved	Decrease of the cyst		3.6	No
11	Open transcallosal fenestration	Complete resolution			0.33	No
12	Open transcortical fenestration	Complete resolution			1	No
13	Stereotactic cysto-ventricular shunt	Marked improvement			1	
14	Stereotactic cysto-peritoneal shunt	Improvement of symptoms				
15	Cystoperitoneal shunt	Died		Died		
16	Endoscopic fenestration, transitory external drainage	Resolution of symptoms, intraventricular hemorrhage	Significant decrease of the cyst	Hemorrhage, external shunting	5	No
17	Endoscopic fenestration, transitory external drainage	Resolution of symptoms	Significant decrease of the cyst		4.5	No
18	Endoscopic fenestration with navigation, transitory external drainage	Resolution of symptoms	Significant decrease of the cyst		4	No
19	Endoscopic fenestration, transitory external drainage	Resolution of symptoms	Significant decrease of the cyst		4	No
20	Endoscopic fenestration with navigation, transitory external drainage	Resolution of symptoms	Significant decrease of the cyst		4	No
21	Endoscopic fenestration, transitory external drainage	Resolution of symptoms	Significant decrease of the cyst		3	No
22	Endoscopic fenestration, transitory external drainage	Resolution of symptoms Significant decrease of the cyst			1.5	No
23	Endoscopic fenestration, transitory external drainage	Resolution of symptoms Significant decrease of the cyst			1.5	No
24	Endoscopic fenestration, transitory external drainage	Resolution of symptoms Significant decrease of the cyst			1	No
25	Endoscopic fenestration, transitory external drainage	Resolution of symptoms Significant decrease of the cyst			1	No
26	Cysto-peritoneal shunt	Resolution of symptoms	Significant decrease of the cyst		1	No
27	Endoscopic fenestration	Resolution of symptoms	Decrease of the cyst		1.5	No
28	Endoscopic fenestration	Decrease of the cyst			1	No
29	Endoscopic fenestration	Resolution of epilepsy	Decrease of the cyst		1.5	No
30	Stereotactic fenestration	Resolution of symptoms	Decrease of the cyst seizure		1	No
31	Endoscopic fenestration	Resolution of symptoms	Decrease of the cyst		0.75	No
32	Endoscopic fenestration	Resolution of symptoms	Decrease of the cyst			
33	Endoscopic fenestration	Resolution of symptoms	Decrease of the cyst		0.5	No
34	Endoscopic fenestration	Resolution of symptoms	Decrease of the cyst		1	No
35	Endoscopic fenestration	Resolution of epilepsy	Decrease of the cyst		1.5	No
36	Endoscopic fenestration (right parietal)	Symptoms unchanged	Decrease of the cyst		0.03	No
37	Endoscopic fenestration with navigation	Resolution of symptoms	Decrease of the cyst		0.25	No
38	Endoscopic fenestration with navigation	Resolution of symptoms	Decrease of the cyst		0.5	No
39	Endoscopic fenestration with navigation	Resolution of symptoms	Decrease of the cyst		0.09	No
40	Endoscopic fenestration with navigation	Resolution of symptoms	Significant decrease of the cyst		5.5	No
41	Endoscopic fenestration with navigation	Resolution of symptoms	Significant decrease of the cyst		7.08	No
42	Endoscopic fenestration with navigation	Resolution of symptoms	Significant decrease of the cyst		5.83	No
43	Endoscopic fenestration with navigation	Symptoms unchanged	Significant decrease of the cyst		4.17	No
44	Endoscopic fenestration with navigation	Resolution of symptoms	Significant decrease of the cyst		2.58	No
45	Endoscopic fenestration with navigation	Complete recovery	Decrease of the cyst		3.5	No
46	Endoscopic fenestration with navigation	Marked improvement	Significant decrease of the cyst		1.75	No
47	Endoscopic fenestration with navigation	Resolution of symptoms	Significant decrease of the cyst		0.5	No
48	Endoscopic fenestration with navigation	Resolution of symptoms	Significant decrease of the cyst		0.25	No
49	Internal shunting	Improvement of symptoms	Significant decrease of the cyst		0.5	No
50	Internal shunting	Resolution of symptoms	Significant decrease of the cyst		3	No
51	Internal shunting	Improvement of symptoms	Significant decrease of the cyst		0.5	No
52	Internal shunting	Improvement of symptoms	Significant decrease of the cyst		3	No
53	Endoscopic fenestration with navigation	Improvement of symptoms	Significant decrease of the cyst		1	No
54	Endoscopic fenestration with navigation	Improvement of symptoms	Significant decrease of the cyst		1	No

## Results

**Fig. 1:**
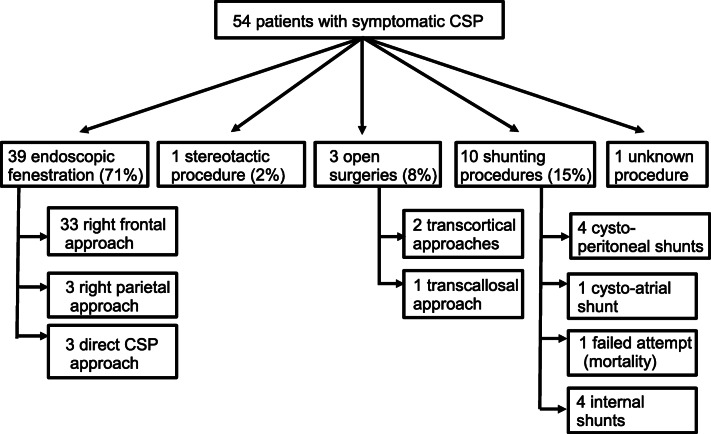
Flowchart showing the different approaches used in the literature review cases

We found 54 cases of symptomatic CSP and CV cysts managed surgically (34 males, 20 females, 1,7/1 male to female ratio). There were 23 children and 21 adults (18 years old). Patients’ characteristics are presented in Table 1. Mean age was 24.3 ± 20.1 years. The most common presentations were headaches (34 patients, 62%), followed by psychiatric symptoms (27 patients, 49.1%). Preoperative radiological hydrocephalus was present in 30 patients (54.5%). Different surgical approaches were performed and are detailed in Fig. 1. The most common surgical procedure was endoscopic fenestration (39patients, 70.9%), followed by shunting (10 patients, 18.2%), open surgery (3 patients, 5.5%), and stereotactic fenestration (1 patient, 1.8%). Complete resolution of symptoms was achieved in 36 patients (65.5%) and partial resolution in 7 patients (13%), and symptoms were unchanged in 2 patients. Complications occurred in 5 patients (9.1%), including 1 death. Recurrence of the cyst occurred in 2 patients (5%). Mean follow-up was 2.8 ± 4.3 months. Comparison between children (*n* = 23) and adults (*n* = 21) revealed statistically significant differences in clinical presentation. There were more headaches in adults (89%) than children (48%), *p* = 0.004. Psychiatric disturbances were more common in children (76%) than adults (33%), *p* = 0.006. The other presenting features did not statistically differ between the two age groups, neither the surgical outcome. Between males (*n* = 34) and females (*n* = 20), only loss of consciousness almost reached significance (only 8% of females, but 33% of males presented with loss of consciousness, *p* = 0.053). Independent predictors of good clinical outcome (resolution or improvement of symptoms) were preoperative radiological hydrocephalus (96% in the good outcome group vs 4% in the bad outcome group, *p* < 0.0001), presence of nausea/vomiting (27% in the good outcome vs 0% in the bad outcome group, *p* = 0.003), and papilledema (13% in the good outcome group, vs 0% in the bad outcome group, *p* = 0.04). Open surgery/shunting procedures (*n* = 13) were associated with poor clinical outcome (*p* = 0.02) compared with endoscopic/stereotactic procedures (*n* = 40). There were no differences between types of endoscopic or stereotactic procedures performed (frontal, parietal, transcavum fenestrations, stereotactic or endoscopic). Recurrence (*n* = 2) was associated with older age (mean = 52 ± 13 years old) than the group without recurrence (*n* = 52) (mean = 22 ± 17 years old), *p* = 0.016. Shunt procedures were also associated with recurrence (50% of recurrence, *p* = 0.027) comparing with the other procedures. Good radiological outcome (decrease of the cyst size) was achieved in all patients, except one (who died). This mortality was attributed to bleeding and infection related to a surgically placed external shunt.

## Discussion

Cavum septum pellucidum and cavum vergae cysts are potential space filled with CSF between the leaflets of the tela choroidea of the third ventricle [1]. As stated in the introduction, CSP is delimited superiorly by the crus of the fornices and inferiorly by the tela choroidea of the third ventricle [1]. CV extends posteriorly to the columns of the fornix. Finally, cavum velum interpositum (CVI) is also anatomically distinct, because it surrounds the internal cerebral veins, whereas CV and CSP lie above them. As CSP and CV cysts were mostly used interchangeably in the literature, we decided to study them together. Usually considered as an incidental finding, and mostly managed conservatively, they may however present with symptoms. Wang et al. [34] found that 22 of 54,000 patients (0.04%) having an MRI had a dilated cyst of the CSP. According to Shaw et al. (1969) [2], cysts may be classified into two groups: incidental (asymptomatic) or pathological (symptomatic). The incidence of symptomatic CSP and CV cysts is hard to define. To the best of our knowledge, the current paper is the first review of the literature concerning symptomatic CSP and CV cysts. Symptomatic CSP and CV cysts are rare and usually present with specific symptoms, such as headaches, behavioral disorders, or cognitive impairment [4]. However, there may be signs and symptoms related to hydrocephalus secondary to the occlusion of the Monroe foramina by the leaflets of the cyst [4, 26]. Moreover, most of the cases reported in the literature presented with radiological evidence of hydrocephalus, as well as regression of the cyst on postoperative imaging^1-^

**Fig. 2:**
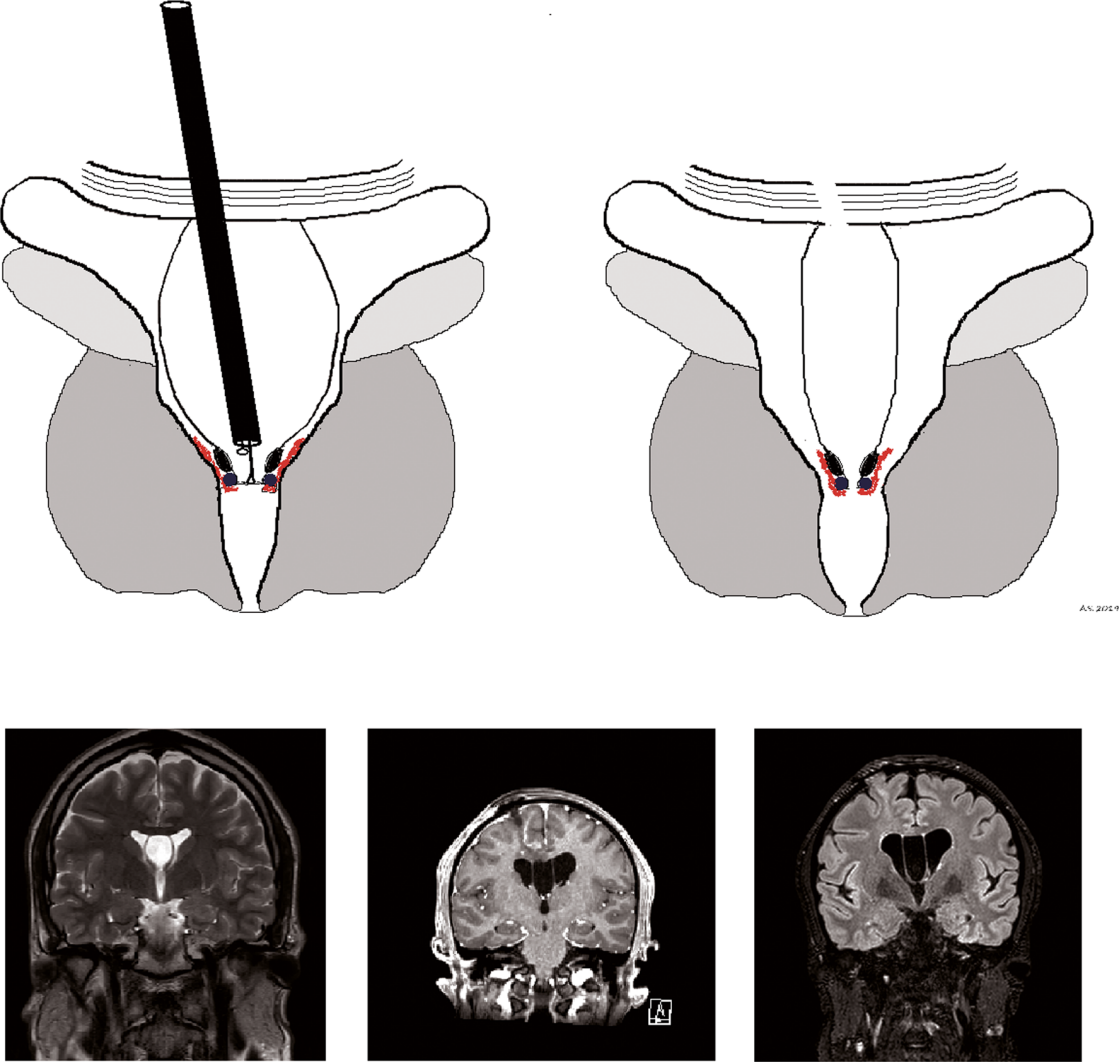
Preoperative (left) and postoperative (right) MRI of a patient with a cavum septum pellucidum cyst fenestrated endoscopically. T2 coronal reconstructions (upper panels) and T1 sagittal reconstructions (lower panels)

[31]. Regarding the size of the cyst, several authors argue that a CSP is defined as a cyst having a width of 10 mm or more between the ventricles [1–5, 19–25]. It may not be reasonable to consider a surgical fenestration for smaller cysts. In our review, the main presenting symptom was headaches (67%), which may be related to the potential implication of hydrocephalus in the development of a symptomatic CSP and CV cyst. Interestingly, psychiatric symptoms, mainly behavioral disturbances, were the second most common finding (56%). We use the generic term “psychiatric symptoms,” because of the heterogeneity of symptoms reported in the literature: behavioral changes, eating disorder, mood disturbances, and anxiety have been reported by several authors [1, 3, 5–10]. These symptoms may be difficult to correlate with CSP and CV cysts. However, an improvement was observed in most of the cases, which may suggest that stretching of midline structures by the cyst could be related to psychiatric disturbances [1]. Our results show that preoperative hydrocephalus was present in most of the patients and was an independent predictor of good clinical outcome. Absence of hydrocephalus at presentation may suggest that the correlation between the cyst and the presenting symptoms is unclear. Conservative management could be offered in those cases, although some reports suggest that symptoms may be improved [1, 3, 5, 6]. Most of the papers reviewed did not specify the duration of symptoms for each patient. However, most of the cases had 1 month to 3 years of symptoms before fenestration [25]. Different procedures have been proposed in the papers we reviewed. The most common approach was endoscopic fenestration of the cyst. This involves a burr-hole craniotomy, most commonly performed in the right frontal region, to fenestrate the cyst to the lateral ventricle. However, other approaches have been described, including parietal cystostomy, or direct transcavum interforniceal endoscopic fenestration, as described by the authors of this manuscript elsewhere (*in press*, Operative Neurosurgery). Preoperative and postoperative images of a patient that benefited from this procedure are illustrated in Fig. 2. Endoscopic and stereotactic approaches may be superior to open or shunting procedures. However, there was no statistically significant difference between the types of endoscopic approach performed regarding the outcome, complications, or recurrence rate. Most of the authors recommend an endoscopic cyst fenestration through a frontal burr-hole, with neuronavigation. Regardless of the technique, most of the cases presented with a reduction of the cyst size after fenestration (Table 2). We advocate a transcavum interforniceal approach to restore more anatomically the flow of CSF to the third ventricle, because it creates a communication between the lateral ventricles and the third ventricle (like the foramen of Monro). Moreover, it avoids midline structures (fornices, internal cerebral veins) that are displaced laterally by the cyst.

## Conclusion

This review of the literature suggests that surgical treatment may be an option for the treatment of symptomatic CSP and CV cysts. Resolution or improvement of the symptoms was achieved in most of the cases. Endoscopic or stereotactic fenestrations seem to be superior to open or shunting procedures, with better clinical outcome and less recurrence. Operative management may be considered for symptomatic CSP and CV cysts, especially when associated with hydrocephalus.
